# Maternal Exposure to Diesel Exhaust Particles (DEPs) During Pregnancy and Adverse Pregnancy Outcomes: Focusing on the Effect of Particulate Matter on Trophoblast, Epithelial-Mesenchymal Transition

**DOI:** 10.3390/cells14171317

**Published:** 2025-08-26

**Authors:** Hyewon Hur, Hayan Kwon, Yun Ji Jung, Euna Choi, Joonggyeong Shin, Subin Jo, Yeji Lee, Min-A Kim, Yong-Sun Maeng, Ja-Young Kwon

**Affiliations:** 1 Department of Obstetrics and Gynecology, Yonsei University College of Medicine, Seoul 03722, Republic of Korea; hwhur201@yuhs.ac (H.H.); whitekwon@yuhs.ac (H.K.); ccstty@yuhs.ac (Y.J.J.);; 2 Department of Obstetrics and Gynecoloty, Institute of Women’s Life Medical Science, Yonsei University Health System, Seoul 03722, Republic of Korea; 3 Department of Medicine, Yonsei University College of Medicine, Seoul 03722, Republic of Korea; 4 Department of Obstetrics and Gynecology, Gangnam Severance Hospital, Yonsei University College of Medicine, Seoul 06273, Republic of Korea; makim302@yuhs.ac

**Keywords:** particulate matter, trophoblast, epithelial-mesenchymal transition (EMT)

## Abstract

During pregnancy, exposure to fine particulate matter (PM_2.5_), particularly diesel exhaust particles (DEPs), elevates the risk of placental dysfunction-related pregnancy complications; however, the underlying cellular mechanisms have yet to be fully elucidated. The objective of this study was to assess the effects of PM_2.5_ exposure on trophoblast functions and their interaction with endometrial stromal cells. We utilized a three-dimensional (3D) model in which human first-trimester trophoblasts (Sw71) formed blastocyst-like spheroids and were cultured with human endometrial stromal cells (HESCs). Trophoblast proliferation, migration, invasion, and 3D network formation following DEP exposure (0.5–20 μg/mL) were assessed using methyl thiazolyl diphenyl-tetrazolium bromide (MTT), wound healing, migration, and invasion assays. The expression levels of genes related to the epithelial-mesenchymal transition (EMT) were quantified by real-time reverse-transcription quantitative polymerase chain reaction (RT-qPCR). DEP exposure significantly inhibited trophoblast proliferation, migration, and invasion. DEP treatment dysregulated the EMT program by significantly decreasing the expression of key mesenchymal markers (*SNAI1*, *SNAI2*, *SOX2*, and *KLF4*) while upregulating epithelial markers. These changes may be related to inhibited trophoblast migration toward HESC monolayers and 3D invasive network formation. DEP directly impairs critical trophoblast functions that are essential for successful pregnancy. Disruption of the EMT program represents a molecular mechanism by which traffic-related air pollution contributes to placental dysfunction and pregnancy complications, highlighting the significant reproductive risks posed by ambient air pollution.

## 1. Introduction

Ambient fine particulate matter (PM_2.5_), poses a significant and escalating global public health concern [1]. The small sizes of PM_2.5_ particles facilitate their deep penetration into the respiratory system, passage across the alveolar–capillary barrier, and subsequent entry into the systemic circulation [2]. These particles transport a diverse array of hazardous chemical components, such as heavy metals, polycyclic aromatic hydrocarbons, and endotoxins [3,4], which in turn can trigger systemic inflammation and oxidative stress [5]. These processes may result in extrapulmonary health complications, such as myocardial infarction [6,7], metabolic disorders [8], neurocognitive dysfunction [9], and inflammatory diseases [5,10]. 

While ambient PM_2.5_ is a heterogeneous mixture of various components, exhaust from diesel engines is a major contributor, particularly in urban settings where population exposure is high [11]. DEPs typically feature a central carbonaceous core with a layer of adsorbed organic compounds on their surface [12]. Diesel exhaust particles (DEPs) are well-established inducers of a range of biological responses, including the synthesis of pro-inflammatory cytokines, the activation of the immune system, and the induction of apoptosis [13,14]. Furthermore, prior research using animal models has shown that prenatal exposure to DEPs can cause several adverse pregnancy outcomes, such as embryo resorption, in utero growth restriction, and pathologic placental morphologies [15,16,17].

Functioning as the interface between the mother and fetus, the placenta is essential both for establishing and for maintaining a successful pregnancy. Thus, proper placental formation is essential for a successful pregnancy, and abnormal placentation may result in pregnancy complications, including miscarriage, preeclampsia, and small for gestational age [18]. Once PM_2.5_ particles enter the systemic circulation, these particles trigger localized immune and cellular inflammatory responses. These responses can induce placental inflammation, which reduces placental transport capacity and the supply of oxygen and nutrients, potentially leading to pregnancy loss, low fetal weight, and preeclampsia [19,20,21,22]. 

In addition to these known cardiorespiratory effects, evidence highlights detrimental effects of PM_2.5_ on human reproductive health. Associations between prenatal exposure to PM_2.5_ and adverse pregnancy outcomes have been demonstrated in numerous epidemiological studies [23,24,25,26]. For instance, direct evidence for the translocation of ambient black carbon particles to the fetal side of the human placenta was provided by the study of Bové et al. [27]. This finding confirms that inhaled PM can be transported toward the maternal–fetal interface and accumulate within placental tissues, thereby posing a direct threat to normal placental development and function. Similarly, in animal studies, PM_2.5_ exposure can result in implantation failure and low birth weight, associated with pathological alterations in placental morphology, including placental hemorrhage, focal necrosis, inflammatory cell infiltration, and oxidative stress [19,28,29,30]. Numerous human epidemiological investigations have reported associations between maternal PM_2.5_ exposure and pregnancy complications, such as preterm delivery [31,32], gestational diabetes mellitus [33,34], preeclampsia [35,36], and recurrent pregnancy loss [37]. At the cellular level, PM_2.5_ exposure induces trophoblast dysfunction, characterized by inhibited proliferation, cell cycle arrest, and diminished migration and invasion capacity in vitro [38,39,40,41]. 

However, despite the informativeness of culture models, these models fail to adequately recapitulate the complex three-dimensional (3D) microenvironment and intricate intercellular signaling of trophoblasts in vivo. In particular, the dynamic and reciprocal communication between invading trophoblasts and maternal endometrial stromal cells, which is a tightly regulated interaction crucial for blastocyst attachment, decidualization, immune tolerance, and spiral artery remodeling, remains a significant yet underexplored area within PM_2.5_ toxicology research [42]. 

Therefore, this study employed a 3D co-culture system integrating human first-trimester trophoblast (Sw71) spheroids with endometrial stromal cells [21] to comprehensively investigate how PM_2.5_ exposure affects trophoblast functions and their intercellular crosstalk with endometrial stromal cells.

## 2. Materials and Methods

### 2.1. Diesel Exhaust Particles

Diesel exhaust particles (DEPs), a significant component of urban PM_2.5_ derived primarily from traffic emissions, were purchased from Sigma-Aldrich Corp., St. Louis, MO, USA (# NIST^®^ SRM^®^ 2975) and produced by light engine combustion. DEPs were weighed using a microbalance (Sartorius, Goettingen, Germany) and suspended in sterile Milli-Q water to yield a stock concentration of 2 mg/mL. The DEPs were then dispersed in the water using a bath-type sonicator (SONICA Soltec; Milan, Italy). The resulting DEP suspension was stored at −20 °C until use. The DEPs were sonicated for 30 min before application to cells and characterized as described previously [43].

### 2.2. Cell Culture

Both human endometrial stromal cells (HESCs) and Sw71 cells were used in our research and characterized as previously reported [44,45]. The Immortalized Human Trophoblast Cell line, hTERT-immortalized (Sw71), was purchased from Applied Biological Materials (abm) Inc. (Cat# T0532, Richmond, BC, Canada). Human Endometrial Stromal Cells (HESCs) were kindly provided by Professor SiHyun Cho from Gangnam Severance Hospital, Yonsei University College of Medicine (Seoul, Republic of Korea). The study protocol was approved by the Institutional Review Board of Gangnam Severance Hospital, Yonsei University College of Medicine (IRB approval no. 3-2021-0336). Informed written consent was obtained from all participants, and the study was conducted in accordance with the principles of the Declaration of Helsinki.

Sw71 cells were cultured in Dulbecco’s modified Eagle’s medium—Nutrient Mixture F-12 (DMEM-F12 medium; Cytiva, Amersham, UK), supplemented with 10% fetal bovine serum (FBS), 10 mmol/L HEPES, 0.1 mmol/L MEM non-essential amino acids, 1 mmol/L sodium pyruvate, and 100 U/mL penicillin/streptomycin(all from GIBCO, MA, USA). HESCs were grown in DMEM supplemented with 10% heat-inactivated FBS, 1.0 mmol/L sodium pyruvate, 10 mmol/L HEPES, 100 mmol/L MEM non-essential amino acids, and 100 U/mL penicillin/streptomycin. All cells were incubated at 37 °C in a 5% CO_2_ atmosphere with 95% humidity.

### 2.3. Cell Proliferation Assay

The proliferation of Sw71 cells was quantified with an MTT (3-(4,5-dimethylthiazol-2yl)-2,5-diphenyltetrazolium bromide) assay, which was conducted according to the protocol provided by the manufacturer(Sigma-Aldrich, St. Louis, MO, USA). Briefly, cells were seeded at a density of 4 × 10^3^ cells/well in a 96-well plate (Corning Inc., Corning, NY, USA). Following seeding in 96-well plates, the trophoblast cells were treated with different DEP concentrations (0.5–20 μg/mL) for 24, 48, and 72 h. Next, the assay was developed by the sequential addition of 15 µL of MTT solution (Sigma-Aldrich) and 200 µL of dimethyl sulfoxide (Sigma-Aldrich) to each well, after which the absorbance was subsequently measured at 490 nm. All groups of experiments were performed in quintuplicate.

### 2.4. Wound-Healing Assay

For the wound-healing assay, we created a scratch in a confluent monolayer of trophoblasts on 35 mm dishes using a micropipette tip. Following the scratch, any loose cells and debris were removed by washing. The cells were subsequently treated with 20 µg/mL of DEPs for a 16 h period. Imaging was performed at the 0 and 16 h time points following the creation of the wound. Quantification was performed by photographing five fields per plate and subsequently the distance between the front lines was measured using ImageJ software (version 1.53t, National Institutes of Health, Bethesda, MD, USA). Each assay was performed in triplicate.

### 2.5. Formation of Blastocyst-like Spheroids (BLS) by Trophoblast Cells

Sw71 cells were trypsinized and cultured in hanging drops (4 × 10^3^ cells/100 μL hanging drop) for 24 or 48 h. Spheroid formation was monitored by examining bright-field images obtained using an Axiovert 200 microscope (Carl Zeiss Micro-Imaging, Thornwood, NY, USA). The following series of in vitro 3D models, including the formation of blastocyst-like spheroids (BLS), 2D migration on endometrial cells, and 3D invasion assays, were established and performed based on the well-characterized protocols previously described by You et al. [21]. To verify that structures emanating from the BLS are trophoblast-derived and to validate the image-analysis workflow, we performed pilot experiments using Sw71 cells constitutively expressing GFP. Live-cell imaging confirmed GFP-positive cords and branches sprouting from the spheroid surface, supporting trophoblast origin of the outgrowth (Supplementary Figure S1).

### 2.6. Two-Dimensional Trophoblast Migration

Single blastocyst-like spheroids (BLSs) were seeded into a 24-well tissue culture plate, with each well containing DMEM-F12 medium with 10% FBS in the presence or absence of DEPs (20 µg/mL). The process of BLS attachment to the well surface and trophoblast migration were observed for 48 h by examining images obtained using an Axiovert 200 microscope. Migration lengths were measured with ImageJ software (version 1.53t, National Institutes of Health, Bethesda, MD, USA).

### 2.7. Two-Dimensional Trophoblast–Endometrial Cell Interactions

For the co-culture assay, a 24-well plate was first prepared with a confluent layer of HESCs in each well. A single BLS was then seeded onto this monolayer, with some wells containing DEPs (20 μg/mL) and others serving as a control. The processes of BLS attachment and their migration onto the cell monolayer were monitored for 72 h via imaging on an Axiovert 200 microscope. Migration lengths were measured with ImageJ software.

### 2.8. Three-Dimensional Trophoblast Invasion and Structure Formation

The endometrium structure was created by seeding HESCs (1 × 10^4^) into flat-bottom 24-well plates and incubating for 24 h. The HESC monolayer was then covered with 300 μL of Matrigel (BD Biosciences, Bedford, MA, USA) that had been diluted 1:1 with 10% FBS in DMEM growth medium. The HESC monolayer was then incubated at 37 °C for 30 min or until the Matrigel had hardened. Then, a single BLS was added onto the surface of the Matrigel layer in 500 μL DMEM-F12 growth medium with or without DEPs (20 μg/mL). To track the invasion and 3D network structure formation of trophoblasts from BLSs, images were recorded at 24 h intervals using an Axiovert 200 microscope. Invasion lengths were measured with ImageJ software (version 1.53t, National Institutes of Health, Bethesda, MD, USA).

### 2.9. Epithelial-Mesenchymal Transition Assay

Sw71 cells were trypsinized and cultured in hanging drops (4 × 10^3^ cells/100 μL hanging drop) for 72 h. Trophoblast monolayer cells cultured in a plate and spheroids in hanging drops (12–72 h) with or without DEPs (20 μg/mL) were harvested with Trizol reagent (Invitrogen, Carlsbad, CA, USA). Real-time reverse-transcription quantitative polymerase chain reaction (RT-qPCR) was employed to quantify gene expression.

### 2.10. RT-qPCR

Total RNA was extracted from the BLS of Sw71 cells using Trizol reagent. The mRNA expression of human genes was measured using a Power SYBR Green RNA-to-CT^TM^ 1-Step kit (Applied Biosystems, Foster City, CA, USA) and StepOnePlus^TM^ (Applied Biosystems), as per the manufacturer’s protocol. The PCR protocol began with a reverse-transcription step at 48 °C for 30 min and an initial denaturation at 95 °C for 10 min. This was followed by 40 cycles, with each cycle consisting of denaturation at 95 °C for 15 s and an annealing/extension step at 55 °C for 1 min. Quantification of the results was based on the obtained cycle threshold (Ct) values. Differences in the Ct values between the experimental and reference (*GAPDH*) genes were calculated, and the results are expressed as the ratio of each RNA level to the calibrated sample. The primers used for gene amplification are shown in Table 1.

### 2.11. Statistical Analyses

All experiments were repeated at least three times, and the data are presented as the mean ± standard error (SE). Statistical significance for comparisons between two groups was determined using the Student’s two-tailed t-test. For comparisons among three or more groups, data were analyzed by one-way or two-way ANOVA followed by Tukey’s post hoc test for multiple comparisons.

## 3. Results

### 3.1. DEPs Decreased the Proliferation and Wound Healing of Sw71 Trophoblasts

**Figure 1:**
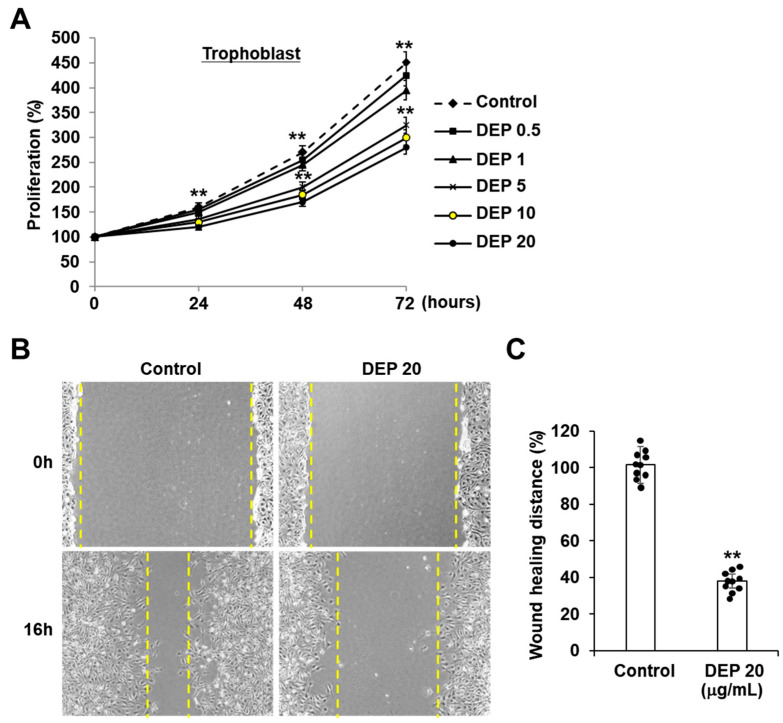
DEPs decreased the proliferation and wound healing of trophoblasts. (**A**) Effect of DEPs on trophoblast proliferation. Trophoblasts were incubated with DEPs (0.5–20 μg/mL) for 72 h, and cell proliferation was assessed using the MTT assay. Statistical significance was determined by two-way repeated measures ANOVA, followed by Tukey’s post hoc test for multiple comparisons. (**B**,**C**) Effect of DEPs on trophoblast wound healing. After creating a scratch in the trophoblasts with a micropipette tip, the culture was treated with DEPs. Imaging was performed at the 0 and 16 h time points following the scratch. Statistical analysis was performed using an unpaired t-test. All data are presented as the mean ± standard error (SE) from three independent experiments performed in duplicate or quintuplicate. ** *p* < 0.01 vs. control.

Sw71 cells were treated with DEPs at varying concentrations to assess the impact on trophoblast function. As shown in Figure 1A, DEPs reduced trophoblast proliferation activity compared to unstimulated cells in a manner dependent on both dose (0.5–20 μg/mL) and time. In addition, the wound-healing assay further confirmed this reduced activity, revealing that fewer Sw71 cells migrated into and closed the cell-free area following DEP treatment (Figure 1B,C). These findings demonstrate that DEPs decrease proliferation and wound healing in Sw71 cells.

### 3.2. DEP Decreased the Migration of Sw71 Trophoblasts

**Figure 2:**
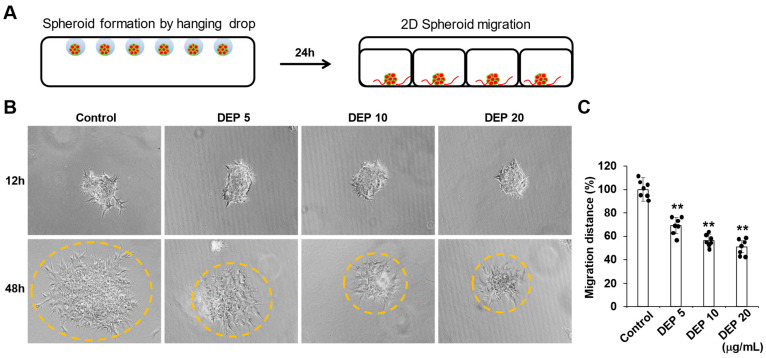
DEPs inhibited the migration of trophoblasts. (**A**) Schematic diagram of the experimental procedure. Trophoblasts (orange circles) were trypsinized and cultured in hanging drops (4 × 10^3^ cells/100 μL hanging drop) for 24 h to form spheroids. BLSs were seeded into individual wells of a 24-well tissue culture plate which contained 10% FBS DMEM-F12 medium with or without DEPs (20 μg/mL) to observe 2D trophoblast migration (represented by orange lines). (**B**) Attachment of BLSs to the well surface and migration of trophoblasts were analyzed by examining images obtained using an Axiovert 200 microscope.(The area of spheroid outgrowth is indicated by the dashed yellow circle.) (**C**) For quantification, the migration distance of every sprout from a single spheroid was measured. Statistical analysis was performed using one-way ANOVA followed by Tukey’s post hoc test for multiple comparisons. All data are presented as the mean ± SE from three different cell lines with *n* = 7 per group per experiment. ** *p* < 0.01 vs. control.

Single blastocyst-like spheroids were generated and then placed into a 24-well tissue culture plate in order to evaluate the impact of DEPs on trophoblast migration (Figure 2A). After BLS transfer, these single 3D BLSs were treated with DEPs, and trophoblast migration was analyzed for 48 h. As shown in Figure 2B, In the control group, trophoblast cells sprouted radially from the entire periphery of the BLS and subsequently migrated across the well surface. However, administering DEPs significantly inhibited the radial sprouting migration of BLS dose-dependently (Figure 2B). We quantified trophoblast migration by using computer-assisted image analysis to measure the distance from the implanted BLSs to the migration edge. (Figure 2C). This result indicates that DEPs decrease the two-dimensional (2D) migration of Sw71 cells.

### 3.3. DEP Reduced the 2D Migration of Sw71 Cells on Endometrial Stromal Cells

**Figure 3:**
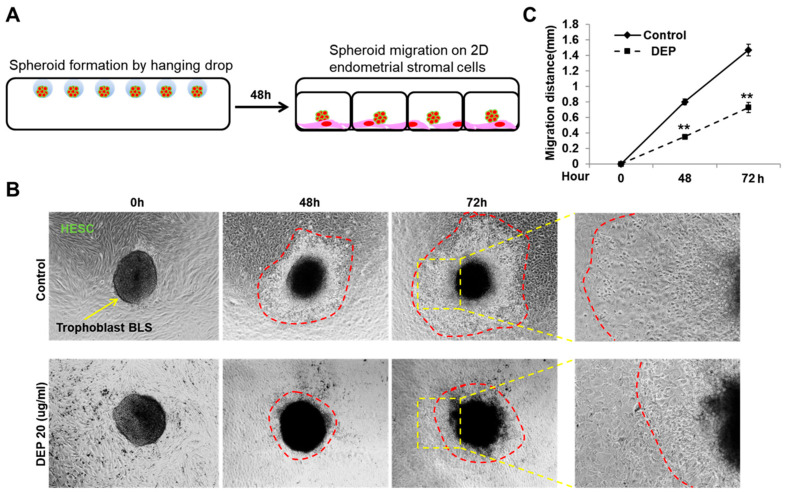
DEPs reduced two-dimensional trophoblast migration on HESCs. (**A**) Trophoblasts were cultured in hanging drops (4 × 10^3^ cells/100 μL hanging drop) for 48 h. We placed single BLSs onto a confluent monolayer of HESCs cultured in a 24-well plate, in the presence or absence of DEPs (20 μg/mL). (**B**) The migration of trophoblasts on HESCs was analyzed over 72 h by examining images obtained using an Axiovert 200 microscope. The yellow arrow indicates the trophoblast BLS. The migration front is delineated by the red dashed line. The area within the yellow dashed box is shown at a higher magnification in the rightmost panel. (**C**) We quantified the migration by measuring the length of all sprouts emerging from each individual spheroid. Statistical significance was determined by two-way repeated measures ANOVA, followed by Tukey’s post hoc test for multiple comparisons. All data are presented as the mean ± SE from three different cell lines, with *n* = 7 per group per experiment. ** *p* < 0.01 vs. control.

The process of implantation involves a critical interaction between invading trophoblasts and two key maternal components, uterine surface epithelium and the endometrial stromal cells [42,46]. A 2D co-culture system was used to examine the impact of DEPs on the interaction between trophoblasts and endometrial cells during implantation (Figure 3A) [21]. As shown in Figure 3B,C, BLS in the control group were transferred into wells containing HESCs and attached to the HESCs before migrating into the stromal cells. However, when the BLSs were transferred into wells containing DEPs, the BLSs attached to HESCs, but the subsequent migration on the endometrial stromal cells decreased. Consequently, these data show that PM_2.5_ reduces the migration of Sw71 cells and may inhibit the specific signals necessary for trophoblasts and endometrial stromal cells to interact during the migration process.

### 3.4. DEPs Inhibited Trophoblast Invasion and 3D Structure Formation in 3D Culture Model with Endometrial Stromal Cells

**Figure 4:**
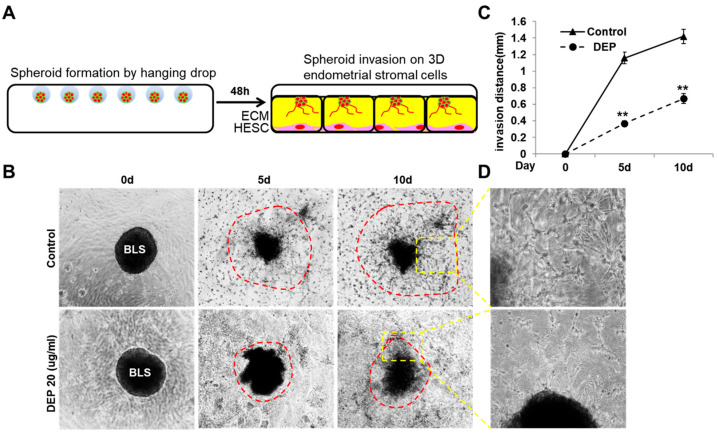
DEPs decreased trophoblast invasion and three-dimensional structure formation in a three-dimensional culture model with HESCs. (**A**) Trophoblasts were cultured in hanging drops (4 × 10^3^ cells/100 μL hanging drop) for 48 h. To serve as a model for the trophectoderm, BLSs were seeded on the surface of the Matrigel layer overlying the HESCs. Invasion of trophoblasts into the Matrigel (**B**) and 3D network formation (**C**) were analyzed over 10 days by examining images obtained using an Axiovert 200 microscope. The invasion front is delineated by the red dashed line. The area within the yellow dahsed box is shown at a higher magnification in the Figure 4D. (**D**) We quantified the invasion by measuring the length of all sprouts emerging from each individual spheroid. Statistical significance was determined by two-way repeated measures ANOVA, followed by Tukey’s post hoc test for multiple comparisons. All data are presented as the mean ± SE from three different cell lines, with *n* = 7 per group per experiment. ** *p* < 0.01 vs. control.

**Figure 5:**
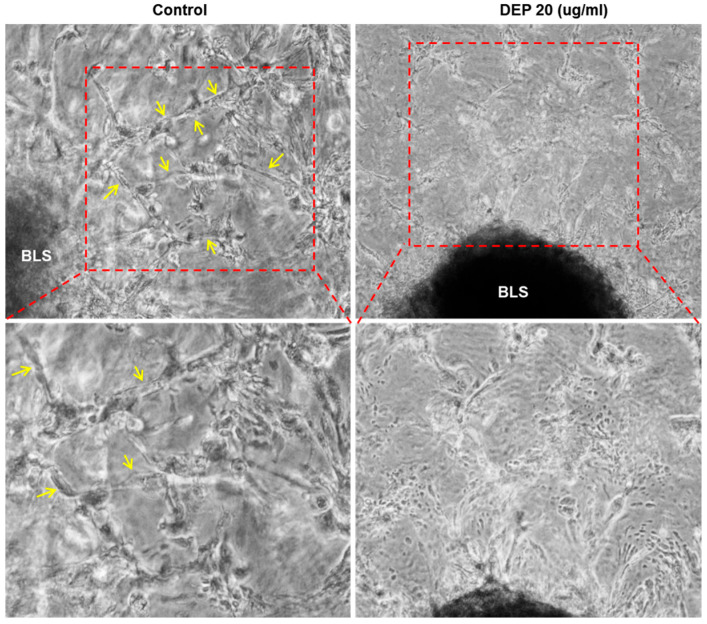
DEPs reduced the number of trophoblasts that emerged from BLSs that had penetrated through the Matrigel and interacted with stromal cells in a 3D model. After 10 days in culture, projections and 3D network formation of trophoblasts within the Matrigel were established. Deep projections of trophoblasts into the Matrigel and their growth toward the stromal cells were analyzed by examining images obtained using an Axiovert 200 microscope. Yellow arrows highlight the deep projections and 3D network formation of invading trophoblasts observed in the control group.

Trophoblast invasion is a dynamic process requiring active migration through uterine tissue layers, including endometrial stroma and extracellular matrix. To facilitate deeper penetration, these trophoblasts release a large amount of proteases that degrade the decidua. However, this process is thought to be regulated, with endometrial stromal cells providing necessary signals to enhance and guide trophoblast invasion [47]. Thus, to better elucidate the effect of DEPs on the crosstalk between stromal cells and invading trophoblasts, we used a 3D in vitro system consisting of Matrigel and HESCs (Figure 4A) [45]. As shown in Figure 4B, in the control group, trophoblast cells were observed to emerge from the BLS and subsequently penetrate the Matrigel layer. However, the addition of DEPs impaired the migratory and invasive activities of the trophoblasts (Figure 4B). To quantify the invasion, the distance between the edge of the implanted BLS and the front of the invading cells was measured using computer-assisted image analysis (Figure 4C). The number of projections and 3D network formation of trophoblasts within the Matrigel were significantly decreased in the DEPs group compared with the control (Figure 4B,D and Figure 5). In addition, deep projections of trophoblasts into the Matrigel and their growth toward the stromal cells were also attenuated following DEP treatment (Figure 5). Collectively, these results suggest that DEPs inhibit trophoblast invasion and 3D network formation toward endometrial stromal cells, which resembles the placental villi interact with the stroma.

### 3.5. DEP Inhibited Epithelial-Mesenchymal Transition

**Figure 6:**
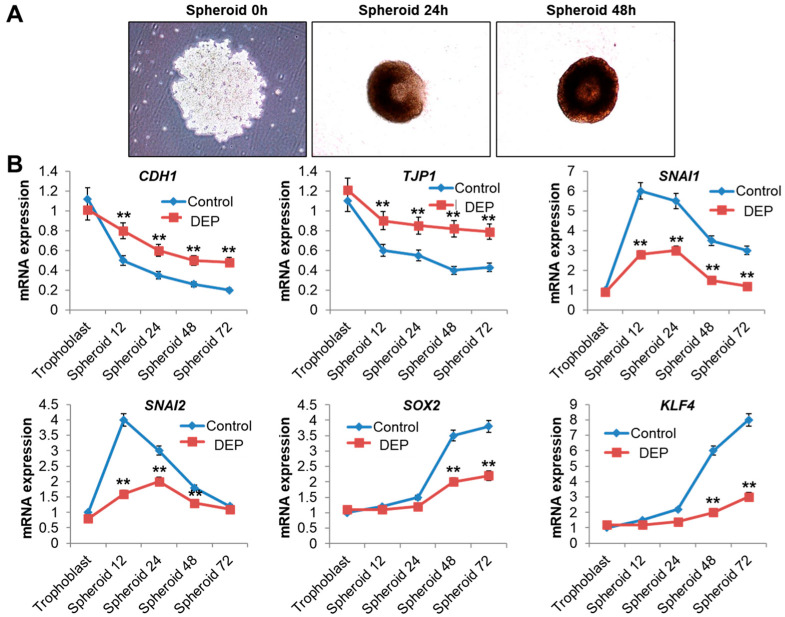
DEP inhibits the expression of genes related to EMT. (**A**) The formation of blastocyst-like spheroids (BLSs). When cultured in a hanging drop, Sw71 trophoblast cells first aggregate, then compact, and ultimately form a round, single 3D spheroid within 48 h. (**B**) RT-qPCR analysis of EMT-related genes. Gene expression was normalized to *GAPDH*. Statistical significance was determined by two-way repeated measures ANOVA, followed by Tukey’s post hoc test for multiple comparisons. Data are presented as mean ± SEM of three independent experiments (*n* = 3). ** *p* < 0.01 vs. control.

Next, we investigated the changes in gene expression that occurred when DEPs were administered during the formation of 3D spheroids. The expression of genes associated with epithelial and mesenchymal phenotypes in Sw71 cells was measured at the mRNA level at various time points during spheroid formation under DEP treatment conditions, as the activation of EMT-related genes confers resistance to anoikis and promotes spheroid formation [48,49]. As shown in Figure 6, trophoblasts expressed epithelial markers, including *CDH1* and *TJP1*, at 0 h. However, these markers were downregulated once the cells were cultured and transferred to a hanging drop, whereas the expression of the mesenchymal genes increased (Figure 6). After the cells had been in low attachment conditions for 12 h, *SNAI1* and *SNAI2* mRNA expression increased considerably, a finding consistent with their known role as early-stage mesenchymal genes. *SOX2* and *KLF4*, which are classified as late mesenchymal genes, exhibited a slower rise and showed significant differences in low attachment conditions after 48 h (Figure 6). However, gene expression change during EMT was inhibited by DEP treatment (Figure 6). These data suggest that DEPs suppress EMT-related gene expression changes during the formation of 3D spheroids.

## 4. Discussion

The objective of this research was to elucidate the effects of DEP exposure on trophoblast functions and their intercellular interactions with endometrial stromal cells.

Using the Sw71 spheroid model, this study demonstrated the direct effects of DEPs in vitro. Specifically, exposure to DEPs resulted in the inhibition of trophoblast proliferation, a pronounced suppression of trophoblast migration and invasion capacity, and a significant dysregulation in the expression of EMT-related genes. Furthermore, our co-culture experiments suggested the potential disruption of trophoblast–decidual crosstalk. 

The exquisite spatiotemporal regulation of trophoblast functions is essential for successful implantation and healthy pregnancy. Trophoblasts proliferate and develop into villous and extravillous trophoblasts (EVTs). The EVTs invade the maternal decidua, ensuring placental anchorage, and profoundly remodel spiral arteries into high-capacitance, low-resistance vessels required for adequate uteroplacental perfusion during gestation. This invasive phenotype is acquired through EMT, conferring migratory and invasive properties [50,51,52,53,54]. Preeclampsia and intrauterine growth restriction (IUGR) are major pregnancy complications associated with trophoblast dysfunction. Both are linked to limited invasion of trophoblasts into the decidua and myometrium, leading to insufficient placentation and poor differentiation. These conditions result in reduced placental function and vascular development problems, hindering the proper placental perfusion and systemic adaptations necessary for a healthy pregnancy [55,56,57].

Our findings indicate that DEP exposure can substantially impair cellular processes indispensable to successful implantation and subsequent placental development, thereby increasing the likelihood of pregnancy complications. The DEP-induced inhibition of trophoblast proliferation observed herein using Sw71 cells corroborates previous findings in other trophoblast lines, such as HTR-8/SVneo, exposed to ambient PM_2.5_ mixtures or specific PM fractions [38,40]. In an in vitro study, Familari et al. treated HTR-8/SVneo trophoblasts with particulate matter from urban environments. They reported that this exposure inhibited cellular growth and changed the protein expression profile. Notably, the alterations in protein expression were similar to patterns observed in preeclampsia and IUGR. The affected proteins were primarily involved in regulating inflammation, endoplasmic reticulum dysfunction, and oxidative stress [40]. Another study further delineated a mechanism involving the activation of the ataxia telangiectasia and Rad3-related protein pathway and G2/M cell cycle arrest underlying PM_2.5_-induced proliferation inhibition [38]. Thus, in agreement with prior research, our findings confirm that the suppression of trophoblast proliferation represents an adverse consequence of PM exposure.

This study also demonstrates that PM exposure during pregnancy impairs the migration and invasion capacity of trophoblasts. Through the processes of migration and invasion, trophoblasts penetrate uterine connective tissues and spiral arteries. These are critical steps for establishing the flow of maternal blood to the embryo [58,59]. Our observations regarding suppressed trophoblast migration and invasion are also consistent with previous in vitro evidence. Qin et al. reported reduced migration and invasion of trophoblasts following PM_2.5_ treatment, which was associated with the upregulation of TIMP1/TIMP2 and downregulation of Collagen I [38]. 

We expanded these previous findings by employing a co-culture system, which demonstrated impaired migration on endometrial stromal cell monolayers and significantly attenuated invasion through Matrigel toward endometrial stromal cells upon exposure to DEPs. This underscores the potential for DEPs to interfere not only with intrinsic trophoblast motility but also with critical paracrine signaling and physical interactions between invading trophoblasts and the receptive endometrium. 

These findings also align with in vivo studies in rodent models, demonstrating that gestational exposure to PM_2.5_ induces pathologic placental alterations, including impaired trophoblast invasion as well as inadequate placental vasculogenesis, ultimately resulting in interference in maternal–fetal interactions and problems in fetal nutrition and growth [60]. For example, recent work by Tosevska et al., employing sophisticated single-cell transcriptomics and flow cytometry in a murine model of intranasal PM exposure (SRM1649b), provided compelling in vivo evidence of pollution-induced alterations to the cellular composition of the placenta. Indeed, Tosevska et al. noted a reduction in specific vascular cell (endothelial and stromal cells) populations and augmented immune cell (NK cells, T-cells, M1/M2 macrophages) infiltration, particularly within the decidua, alongside an enrichment of transcripts associated with inflammatory pathways [61].

Soto et al. exposed one group to PM_2.5_ and kept another group as a control, and reported decreased placental expression of TGF-β1 in the group exposed to PM_2.5_ [62]. TGF-β1 is a critical factor that facilitates the successful invasion of trophoblasts into the maternal vasculature during implantation [63]. A significant reduction was also observed in the levels of VEGF-A, a primary regulator of vascular structure formation, in the group exposed to PM_2.5_ [62,64]. Based on these findings, PM_2.5_ exposure during pregnancy not only impairs trophoblast invasion and differentiation but also leads to deficient placental vascularization, potentially resulting in implantation failure and placenta-mediated pregnancy complications. 

We demonstrated that DEP exposure dysregulates the expression of EMT-associated genes in human trophoblasts, which represents a significant finding. Moreover, this observation highlights a potentially critical, yet underappreciated, molecular mechanism through which traffic-derived air pollution may impair placental development.

EMT constitutes a fundamental cellular reprogramming process essential for embryonic development and tissue remodeling. During implantation and placentation, villous cytotrophoblasts undergo a regulated EMT to differentiate into invasive EVTs. This transition entails the loss of epithelial and the acquisition of mesenchymal attributes, enabling EVTs to migrate through the decidua, invade maternal spiral arteries, and ultimately establish adequate feto-maternal circulation [51,52,65].

Precise regulation of trophoblast EMT is crucial for a successful pregnancy. Thus, impairment of this process, culminating in shallow placental invasion, constitutes a hallmark of severe pregnancy complications such as preeclampsia and IUGR [54,55]. Indeed, studies examining placentas from patients with preeclampsia have revealed altered expression profiles of EMT markers, including increased E-cadherin and decreased vimentin expression, indicative of defective EMT [56,65]. Consequently, factors disrupting trophoblast EMT pose a potential risk to gestational health.

A central implication of our data is that DEPs attenuate the trophoblast EMT program, highlighting a critical molecular mechanism through which traffic-derived air pollution may impair placental development. In Sw71 spheroids under control conditions, we observed a canonical trajectory—an early decline in epithelial markers (*CDH1, TJP1*), induction of EMT-initiating transcription factors (*SNAI1/2*), and a later increase in factors associated with mesenchymal/stem-like states (*SOX2, KLF4*). By contrast, DEP exposure flattened this sequence, preserving epithelial identity while markedly blunting the expected induction of mesenchymal markers, *SNAI1/2, SOX2, and KLF4*. This molecular brake provides a parsimonious explanation for the functional phenotypes observed across our 2D/3D assays—reduced migration and invasion and impaired 3D network formation. Because extravillous trophoblast differentiation and uterine invasion are EMT-dependent during normal implantation, derailment of this program offers a plausible mechanistic axis linking traffic-related particulate exposure to shallow placentation and placenta-mediated complications such as preeclampsia and fetal growth restriction. Although the present readouts are at the transcript level, future studies should verify protein abundance and subcellular localization and delineate the upstream pathways through which DEP perturbs EMT.

Although it is demonstrated that exposure to PM_2.5_ induces EMT-like alterations in other biological contexts, such as bronchial epithelial cells, often mediated by reactive oxygen species (ROS), TGF-β, or inflammatory signaling [30,66,67], few studies have directly investigated the impact of specific air pollutants, such as DEPs, on trophoblast EMT [68]. We believe that this study offers the first experimental evidence that DEPs directly interfere with EMT-related gene expression in human trophoblasts. This finding offers a specific molecular insight into the mechanisms through which traffic-related air pollution may exert reproductive toxicity, specifically through the disruption of a key cellular differentiation pathway essential for placentation. However, our conclusions regarding the EMT pathway are based on robust gene expression data, but these transcriptional changes were not confirmed at the protein level. This lack of protein analysis is a key limitation, and confirming these findings is a critical direction for future research.

The mechanisms through which PM affects trophoblasts are complex and likely encompass multiple interacting pathways. Oxidative stress, elicited by ROS generation upon interaction with PM constituents, represents a central mechanism implicated in numerous studies [4,69], resulting in downstream cellular damage, including DNA damage [70,71] and mitochondrial dysfunction [72,73]. Meanwhile, placental inflammation, potentially initiated by direct PM interaction or systemic inflammatory signaling, represents another key pathway [19,68,74,75], a notion supported by evidence of increased inflammatory markers and immune cell infiltration in vivo [61,76,77]. Epigenetic modifications [78], endothelial dysfunction [5,62,79], and altered coagulation cascades [80] have also been posited. Our identification of EMT dysregulation introduces an additional dimension to this framework, potentially representing a downstream consequence of, or a pathway that interacts with, these established mechanisms.

Within urban environments, a significant fraction of PM_2.5_ originates from traffic emissions, with DEPs representing a major and highly toxic component [81]. DEPs, rich in PAHs, heavy metals, and endotoxins, are known to trigger systemic inflammation and oxidative stress [3,4,5]. Although determining the precise placental PM dose in vivo following maternal inhalation remains challenging, the DEP concentrations employed in this study (ranging up to 20 µg/mL) were selected to represent concentrations potentially relevant to human environmental exposures. The concentrations used in our study (10 and 20 µg/mL) are consistent with the dose ranges (10–100 µg/mL) used in previous in vitro studies investigating the effects of particulate matter on trophoblast function [38,82,83]. Lower concentrations (e.g., up to 1 µg/mL) may approximate ambient PM_2.5_ concentrations near the short-term guideline values recommended by the World Health Organization (approx. 5 µg/m^3^), while higher concentrations might simulate exposures encountered in heavily polluted urban environments where PM_2.5_ levels may exceed 100 µg/m^3^ [41]. Nevertheless, caution is warranted when extrapolating in vitro dosimetry to real-world human exposure scenarios, owing to disparities in exposure routes, particle transformation, biodistribution, and clearance. 

We acknowledge that the use of the MTT assay is a limitation of our study for assessing proliferation. As the MTT assay measures metabolic activity, the results reflect changes in cell viability and not necessarily a direct inhibition of cell division. Future studies should employ more direct methods, such as DNA content measurement, to confirm these findings.

This study experimentally elucidated the direct impact of DEPs, an environmentally relevant pollutant, on multiple crucial trophoblast functions using validated assays within a 3D co-culture system using Sw71 spheroids. The Sw71 cell line, when cultured under low attachment conditions, forms BLSs that recapitulate key features of early trophoblast development, including compaction, EMT marker expression, and the acquisition of an EVT-like phenotype [21,44,84]. Therefore, this system enables the robust evaluation of trophoblast migration, invasion, and crosstalk with endometrial stromal cells within an ECM-like environment. Furthermore, the physiological relevance, reproducibility, and ability to mimic early implantation events of this system make it a superior alternative to conventional 2D cultures for studying environmental toxicology of the maternal–fetal interface [84]. While our study provides valuable insights into the effects of DEPs on trophoblasts, we must acknowledge several limitations related to our in vitro model and study scope. Our study utilized the Sw71 cell line, which was originally immortalized and characterized as having a stable extravillous trophoblast (EVT) phenotype, expressing key markers such as Cytokeratin 7, Vimentin, and HLA-G [21,84]. The validity of this cell line as a reliable surrogate for primary human trophoblasts in 3D spheroid models, which was a central part of our investigation, has been further reinforced by a recent direct validation study concluding that they are interchangeable in terms of phenotype and function [84].

Based on this well-characterized cell model, our interpretation of the invasive projections in our 3D assay was based on clear morphological evidence, which is highly consistent with the methodology first established by G. Mor et al. [21]. We acknowledge that our morphological assessment, while consistent with previous reports, has inherent limitations. We acknowledge that without definitive in-situ immunofluorescence to specifically identify cell types, the cellular origin of all cells within these projections cannot be unequivocally confirmed. These aspects are important limitations and represent crucial directions for future research.

At the same time, the 3D experiments conducted do not permit definitive subclassification of sprouting trophoblasts as cytotrophoblast (CT) versus extravillous trophoblast (EVT). In our hands, immunophenotyping within Matrigel was limited by antibody penetration and specificity, and under DEP exposure lineage-marker signals were insufficiently robust for unambiguous calls. Moreover, the extent to which Sw71 cells fully recapitulate CT to EVT differentiation under a given 3D condition remains context-dependent. For these reasons we refrained from over-interpreting lineage identity and focused on quantitative motility/invasion readouts. Future studies using optimized 3D immunolabeling/clearing, reporter systems (e.g., HLA-G reporters), or single-cell transcriptomic profiling will be valuable to resolve CT versus EVT identity under DEP.

Additionally, identification of EMT dysregulation offers novel molecular insights into the potential mechanisms underlying PM-induced reproductive toxicity. Although this study identified EMT-related gene expression dysregulation, it did not delineate the specific upstream signaling pathways mediating this effect. Moreover, while our co-culture model allowed for the observation of trophoblast behavior in the presence of HESCs, a more detailed characterization of the bidirectional molecular signals constituting crosstalk and an investigation into how DEPs perturb these signals are required. 

Future investigations should aim to dissect the specific molecular pathways (e.g., NF-κB, ROS-dependent cascades) [47,50,85,86] through which DEPs alter the expression and activity of key EMT-regulating transcription factors in trophoblasts [53,54,67,87,88]. Moreover, further research exploring the interplay between DEP-induced EMT disruption and other cellular stress responses is warranted. Elucidating the specific molecular signals (e.g., cytokines, growth factors, extracellular vesicles) involved in trophoblast—decidual communication that are perturbed by DEP exposure is a critical next step. 

## 5. Conclusions

This study provides compelling evidence that DEP exposure directly impairs key human trophoblast functions, including proliferation, migration, and invasion, and, critically, dysregulates the EMT program essential for placental development in vitro. Furthermore, our findings using a 3D co-culture model suggest potential adverse effects on vital trophoblast–decidual communication. These results delineate plausible cellular and molecular mechanisms contributing to the reproductive toxicity associated with traffic-related air pollution, offering a deeper insight into the mechanisms by which PM exposure during early pregnancy results in adverse outcomes. This research underscores the critical importance of developing strategies to mitigate air pollution and protect feto-maternal health.

## Figures and Tables

**Table 1:** Primer sequences for RT-qPCR.

*CDH1*	F-aaagcctcaggtcataaaca	*S* *OX2*	F-ccacctacagcatgtcctac
	R-gttgggtcgttgtactgaat		R-gagtgggaggaagaggtaac
*TJP1*	F-gagcacatggtgaaggtaat	*KLF4*	F-ggcaaaacctacacaaagag
	R-ctgaaagttgctggcttatt		R-gtagtgcctggtcagttcat
*S* *NAI1*	F-cttcagtctcttccttggag	*GAPDH*	F-atggggaaggtgaaggtcg
	R-gttgcagtatttgcagttga		R-ggggtcattgatggcaacaata
*SNAI2*	F-tgtcataccacaaccagaga		
	R-agtatccggaaagaggagag		

## Data Availability

All data included in this study are available upon request by contact with the first author or corresponding author.

## Supplementary Materials

The following supporting information can be downloaded at: https://www.mdpi.com/article/10.3390/cells14171317/s1, Figure S1: Validation of trophoblast origin of 3D outgrowth using GFP-expressing Sw71 cells. Constitutive GFP expression confirms that cords and branches emerging from the spheroid surface are trophoblast-derived. Phase-contrast and fluorescence channels are shown; images were acquired under identical conditions to those used for quantification in the main figures.

## Abbreviations

The following abbreviations are used in this manuscript:

DEP	Diesel exhaust particles
PM_2.5_	Fine particulate matter
HESC	Human endometrial stromal cell
Sw71	Human first-trimester trophoblasts swan cell
EMT	Epithelial-mesenchymal transition

## References

[1] Agency, U.S.E.P. Particulate Matter (PM) Basics. https://www.epa.gov/pm-pollution/particulate-matter-pm-basics.

[2] Nemmar A., Hoet P.H., Vanquickenborne B., Dinsdale D., Thomeer M., Hoylaerts M.F., Vanbilloen H., Mortelmans L., Nemery B. (2002). Passage of inhaled particles into the blood circulation in humans. Circulation.

[3] Feng S., Gao D., Liao F., Zhou F., Wang X. (2016). The health effects of ambient PM_2.5_ and potential mechanisms. Ecotoxicol. Environ. Saf..

[4] Jan R., Roy R., Bhor R., Pai K., Satsangi P.G. (2020). Toxicological screening of airborne particulate matter in atmosphere of pune: Reactive oxygen species and cellular toxicity. Environ. Pollut..

[5] Pope C.A., Bhatnagar A., McCracken J.P., Abplanalp W., Conklin D.J., O’Toole T. (2016). Exposure to fine particulate air pollution is associated with endothelial injury and systemic inflammation. Circ. Res..

[6] Olaniyan T., Pinault L., Li C., van Donkelaar A., Meng J., Martin R.V., Hystad P., Robichaud A., Ménard R., Tjepkema M. (2022). Ambient air pollution and the risk of acute myocardial infarction and stroke: A national cohort study. Environ. Res..

[7] Farhadi Z., Abulghasem Gorgi H., Shabaninejad H., Aghajani Delavar M., Torani S. (2020). Association between PM_2.5_ and risk of hospitalization for myocardial infarction: A systematic review and a meta-analysis. BMC Public Health.

[8] Wallwork R.S., Colicino E., Zhong J., Kloog I., Coull B.A., Vokonas P., Schwartz J.D., Baccarelli A.A. (2017). Ambient fine particulate matter, outdoor temperature, and risk of metabolic syndrome. Am. J. Epidemiol..

[9] Carey I.M., Anderson H.R., Atkinson R.W., Beevers S.D., Cook D.G., Strachan D.P., Dajnak D., Gulliver J., Kelly F.J. (2018). Are noise and air pollution related to the incidence of dementia? A cohort study in london, england. BMJ Open.

[10] Rezayat A.A., Niloufar J., Mir Nourbakhsh S.H., Hasheminezhad Hoseini F.S., Hooshmand N., Ghasemi Nour M., Handjani F., Tabrizi R. (2022). The effect of air pollution on systemic lupus erythematosus: A systematic review and meta-analysis. Lupus.

[11] Lewtas J. (2007). Air pollution combustion emissions: Characterization of causative agents and mechanisms associated with cancer, reproductive, and cardiovascular effects. Mutat. Res..

[12] Wang B., Lau Y.S., Huang Y., Organ B., Chuang H.C., Ho S.S.H., Qu L., Lee S.C., Ho K.F. (2021). Chemical and toxicological characterization of particulate emissions from diesel vehicles. J. Hazard. Mater..

[13] Hiura T.S., Kaszubowski M.P., Li N., Nel A.E. (1999). Chemicals in diesel exhaust particles generate reactive oxygen radicals and induce apoptosis in macrophages. J. Immunol..

[14] Gibbs J.L., Dallon B.W., Lewis J.B., Walton C.M., Arroyo J.A., Reynolds P.R., Bikman B.T. (2019). Diesel exhaust particle exposure compromises alveolar macrophage mitochondrial bioenergetics. Int. J. Mol. Sci..

[15] Valentino S.A., Tarrade A., Aioun J., Mourier E., Richard C., Dahirel M., Rousseau-Ralliard D., Fournier N., Aubrière M.C., Lallemand M.S. (2016). Maternal exposure to diluted diesel engine exhaust alters placental function and induces intergenerational effects in rabbits. Part. Fibre Toxicol..

[16] Bongaerts E., Nawrot T.S., Wang C., Ameloot M., Bové H., Roeffaers M.B., Chavatte-Palmer P., Couturier-Tarrade A., Cassee F.R. (2023). Placental-fetal distribution of carbon particles in a pregnant rabbit model after repeated exposure to diluted diesel engine exhaust. Part. Fibre Toxicol..

[17] Thaver S., Foa L., Richards S.M., Lyons A.B., Zosky G.R. (2021). In utero exposure to diesel exhaust particles, but not silica, alters post-natal immune development and function. Chemosphere.

[18] Ma X.L., Li X., Tian F.J., Zeng W.H., Zhang J., Mo H.Q., Qin S., Sun L.Q., Zhang Y.C., Zhang Y. (2020). Upregulation of rnd3 affects trophoblast proliferation, apoptosis, and migration at the maternal-fetal interface. Front. Cell Dev. Biol..

[19] Liu Y., Wang L., Wang F., Li C. (2016). Effect of fine particulate matter (PM_2.5_) on rat placenta pathology and perinatal outcomes. Med. Sci. Monit..

[20] González-Comadran M., Jacquemin B., Cirach M., Lafuente R., Cole-Hunter T., Nieuwenhuijsen M., Brassesco M., Coroleu B., Checa M.A. (2021). The effect of short term exposure to outdoor air pollution on fertility. Reprod. Biol. Endocrinol..

[21] You Y., Stelzl P., Zhang Y., Porter J., Liu H., Liao A.H., Aldo P.B., Mor G. (2019). Novel 3d in vitro models to evaluate trophoblast migration and invasion. Am. J. Reprod. Immunol..

[22] Tapia V.L., Vasquez B.V., Vu B., Liu Y., Steenland K., Gonzales G.F. (2020). Association between maternal exposure to particulate matter (PM_2.5_) and adverse pregnancy outcomes in lima, peru. J. Expo. Sci. Environ. Epidemiol..

[23] Sun M., Yan W., Fang K., Chen D., Liu J., Chen Y., Duan J., Chen R., Sun Z., Wang X. (2020). The correlation between PM_2.5_ exposure and hypertensive disorders in pregnancy: A meta-analysis. Sci. Total Environ..

[24] Pedersen M., Stayner L., Slama R., Sørensen M., Figueras F., Nieuwenhuijsen M.J., Raaschou-Nielsen O., Dadvand P. (2014). Ambient air pollution and pregnancy-induced hypertensive disorders: A systematic review and meta-analysis. Hypertension.

[25] Rammah A., Whitworth K.W., Han I., Chan W., Symanski E. (2019). PM_2.5_ metal constituent exposure and stillbirth risk in harris county, texas. Environ. Res..

[26] Michikawa T., Morokuma S., Yamazaki S., Takami A., Sugata S., Yoshino A., Takeda Y., Nakahara K., Saito S., Hoshi J. (2022). Exposure to chemical components of fine particulate matter and ozone, and placenta-mediated pregnancy complications in tokyo: A register-based study. J. Expo. Sci. Environ. Epidemiol..

[27] Bové H., Bongaerts E., Slenders E., Bijnens E.M., Saenen N.D., Gyselaers W., Van Eyken P., Plusquin M., Roeffaers M.B.J., Ameloot M. (2019). Ambient black carbon particles reach the fetal side of human placenta. Nat. Commun..

[28] Enquobahrie D.A., MacDonald J., Hussey M., Bammler T.K., Loftus C.T., Paquette A.G., Byington N., Marsit C.J., Szpiro A., Kaufman J.D. (2022). Prenatal exposure to particulate matter and placental gene expression. Environ. Int..

[29] Brunst K.J., Sanchez-Guerra M., Chiu Y.M., Wilson A., Coull B.A., Kloog I., Schwartz J., Brennan K.J., Bosquet Enlow M., Wright R.O. (2018). Prenatal particulate matter exposure and mitochondrial dysfunction at the maternal-fetal interface: Effect modification by maternal lifetime trauma and child sex. Environ. Int..

[30] Chi Y., Huang Q., Lin Y., Ye G., Zhu H., Dong S. (2018). Epithelial-mesenchymal transition effect of fine particulate matter from the yangtze river delta region in china on human bronchial epithelial cells. J. Environ. Sci..

[31] Kirwa K., Feric Z., Manjourides J., Alshawabekeh A., Vega C.M.V., Cordero J.F., Meeker J.D., Suh H.H. (2021). Preterm birth and PM_2.5_ in puerto rico: Evidence from the protect birth cohort. Environ. Health.

[32] Alman B.L., Stingone J.A., Yazdy M., Botto L.D., Desrosiers T.A., Pruitt S., Herring A.H., Langlois P.H., Nembhard W.N., Shaw G.M. (2019). Associations between PM_2.5_ and risk of preterm birth among liveborn infants. Ann. Epidemiol..

[33] Miron-Celis M., Talarico R., Villeneuve P.J., Crighton E., Stieb D.M., Stanescu C., Lavigne É. (2023). Critical windows of exposure to air pollution and gestational diabetes: Assessing effect modification by maternal pre-existing conditions and environmental factors. Environ. Health.

[34] Cheng X., Ji X., Yang D., Zhang C., Chen L., Liu C., Meng X., Wang W., Li H., Kan H. (2022). Associations of PM_2.5_ exposure with blood glucose impairment in early pregnancy and gestational diabetes mellitus. Ecotoxicol. Environ. Saf..

[35] Weber K.A., Yang W., Lurmann F., Hammond S.K., Shaw G.M., Padula A.M. (2019). Air pollution, maternal hypertensive disorders, and preterm birth. Environ. Epidemiol..

[36] Savitz D.A., Elston B., Bobb J.F., Clougherty J.E., Dominici F., Ito K., Johnson S., McAlexander T., Ross Z., Shmool J.L. (2015). Ambient fine particulate matter, nitrogen dioxide, and hypertensive disorders of pregnancy in new york city. Epidemiology.

[37] Gaskins A.J., Hart J.E., Chavarro J.E., Missmer S.A., Rich-Edwards J.W., Laden F., Mahalingaiah S. (2019). Air pollution exposure and risk of spontaneous abortion in the nurses’ health study ii. Hum. Reprod..

[38] Qin Z., Hou H., Fu F., Wu J., Han B., Yang W., Zhang L., Cao J., Jin X., Cheng S. (2017). Fine particulate matter exposure induces cell cycle arrest and inhibits migration and invasion of human extravillous trophoblast, as determined by an itraq-based quantitative proteomics strategy. Reprod. Toxicol..

[39] Raez-Villanueva S., Ma C., Kleiboer S., Holloway A.C. (2018). The effects of electronic cigarette vapor on placental trophoblast cell function. Reprod. Toxicol..

[40] Familari M., Nääv Å., Erlandsson L., de Iongh R.U., Isaxon C., Strandberg B., Lundh T., Hansson S.R., Malmqvist E. (2019). Exposure of trophoblast cells to fine particulate matter air pollution leads to growth inhibition, inflammation and er stress. PLoS ONE.

[41] Nääv Å., Erlandsson L., Isaxon C., Åsander Frostner E., Ehinger J., Sporre M.K., Krais A.M., Strandberg B., Lundh T., Elmér E. (2020). Urban PM_2.5_ induces cellular toxicity, hormone dysregulation, oxidative damage, inflammation, and mitochondrial interference in the hrt8 trophoblast cell line. Front. Endocrinol..

[42] Gellersen B., Wolf A., Kruse M., Schwenke M., Bamberger A.M. (2013). Human endometrial stromal cell-trophoblast interactions: Mutual stimulation of chemotactic migration and promigratory roles of cell surface molecules cd82 and ceacam1. Biol. Reprod..

[43] Bengalli R., Zerboni A., Marchetti S., Longhin E., Priola M., Camatini M., Mantecca P. (2019). In vitro pulmonary and vascular effects induced by different diesel exhaust particles. Toxicol. Lett..

[44] Straszewski-Chavez S.L., Abrahams V.M., Alvero A.B., Aldo P.B., Ma Y., Guller S., Romero R., Mor G. (2009). The isolation and characterization of a novel telomerase immortalized first trimester trophoblast cell line, swan 71. Placenta.

[45] Krikun G., Mor G., Alvero A., Guller S., Schatz F., Sapi E., Rahman M., Caze R., Qumsiyeh M., Lockwood C.J. (2004). A novel immortalized human endometrial stromal cell line with normal progestational response. Endocrinology.

[46] Aplin J.D., Ruane P.T. (2017). Embryo-epithelium interactions during implantation at a glance. J. Cell Sci..

[47] Velicky P., Knofler M., Pollheimer J. (2016). Function and control of human invasive trophoblast subtypes: Intrinsic vs. Maternal control. Cell Adh Migr..

[48] Cao Z., Livas T., Kyprianou N. (2016). Anoikis and emt: Lethal “liaisons” during cancer progression. Crit. Rev. Oncog..

[49] Paoli P., Giannoni E., Chiarugi P. (2013). Anoikis molecular pathways and its role in cancer progression. Biochim. Biophys. Acta.

[50] Lunghi L., Ferretti M.E., Medici S., Biondi C., Vesce F. (2007). Control of human trophoblast function. Reprod. Biol. Endocrinol..

[51] DaSilva-Arnold S.C., Zamudio S., Al-Khan A., Alvarez-Perez J., Mannion C., Koenig C., Luke D., Perez A.M., Petroff M., Alvarez M. (2018). Human trophoblast epithelial-mesenchymal transition in abnormally invasive placenta†. Biol. Reprod..

[52] Kalluri R., Weinberg R.A. (2009). The basics of epithelial-mesenchymal transition. J. Clin. Investig..

[53] Oghbaei F., Zarezadeh R., Jafari-Gharabaghlou D., Ranjbar M., Nouri M., Fattahi A., Imakawa K. (2022). Epithelial-mesenchymal transition process during embryo implantation. Cell Tissue Res..

[54] Davies J.E., Pollheimer J., Yong H.E.J., Kokkinos M.I., Kalionis B., Knöfler M., Murthi P. (2016). Epithelial-mesenchymal transition during extravillous trophoblast differentiation. Cell Adh Migr..

[55] Lim K.H., Zhou Y., Janatpour M., McMaster M., Bass K., Chun S.H., Fisher S.J. (1997). Human cytotrophoblast differentiation/invasion is abnormal in pre-eclampsia. Am. J. Pathol..

[56] Ge H., Yin N., Han T.L., Huang D., Chen X., Xu P., He C., Tong C., Qi H. (2019). Interleukin-27 inhibits trophoblast cell invasion and migration by affecting the epithelial-mesenchymal transition in preeclampsia. Reprod. Sci..

[57] Cui J., Yang Z., Ma R., He W., Tao H., Li Y., Zhao Y. (2024). Placenta-targeted treatment strategies for preeclampsia and fetal growth restriction: An opportunity and major challenge. Stem Cell Rev. Rep..

[58] Huppertz B. (2019). Traditional and new routes of trophoblast invasion and their implications for pregnancy diseases. Int. J. Mol. Sci..

[59] van den Brûle F., Berndt S., Simon N., Coulon C., Le Goarant J., Munaut C., Noël A., Frankenne F., Foidart J.M. (2005). Trophoblast invasion and placentation: Molecular mechanisms and regulation. Chem. Immunol. Allergy.

[60] Yue H., Ji X., Zhang Y., Li G., Sang N. (2019). Gestational exposure to PM_2.5_ impairs vascularization of the placenta. Sci. Total Environ..

[61] Tosevska A., Ghosh S., Ganguly A., Cappelletti M., Kallapur S.G., Pellegrini M., Devaskar S.U. (2022). Integrated analysis of an in vivo model of intra-nasal exposure to instilled air pollutants reveals cell-type specific responses in the placenta. Sci. Rep..

[62] Soto S.F., Melo J.O., Marchesi G.D., Lopes K.L., Veras M.M., Oliveira I.B., Souza R.M., de Castro I., Furukawa L.N.S., Saldiva P.H.N. (2017). Exposure to fine particulate matter in the air alters placental structure and the renin-angiotensin system. PLoS ONE.

[63] Lafontaine L., Chaudhry P., Lafleur M.J., Van Themsche C., Soares M.J., Asselin E. (2011). Transforming growth factor beta regulates proliferation and invasion of rat placental cell lines. Biol. Reprod..

[64] Shibuya M. (2013). Vascular endothelial growth factor and its receptor system: Physiological functions in angiogenesis and pathological roles in various diseases. J. Biochem..

[65] Zuo Q., Huang S., Zou Y., Xu Y., Jiang Z., Zou S., Xu H., Sun L. (2016). The lnc rna spry4-it1 modulates trophoblast cell invasion and migration by affecting the epithelial-mesenchymal transition. Sci. Rep..

[66] Wei H., Liang F., Cheng W., Zhou R., Wu X., Feng Y., Wang Y. (2017). The mechanisms for lung cancer risk of PM_2.5_: Induction of epithelial-mesenchymal transition and cancer stem cell properties in human non-small cell lung cancer cells. Environ. Toxicol..

[67] Xu Z., Ding W., Deng X. (2019). PM_2.5_, fine particulate matter: A novel player in the epithelial-mesenchymal transition?. Front. Physiol..

[68] de Melo J.O., Soto S.F., Katayama I.A., Wenceslau C.F., Pires A.G., Veras M.M., Furukawa L.N., de Castro I., Saldiva P.H., Heimann J.C. (2015). Inhalation of fine particulate matter during pregnancy increased il-4 cytokine levels in the fetal portion of the placenta. Toxicol. Lett..

[69] Wang B., Chan Y.L., Li G., Ho K.F., Anwer A.G., Smith B.J., Guo H., Jalaludin B., Herbert C., Thomas P.S. (2021). Maternal particulate matter exposure impairs lung health and is associated with mitochondrial damage. Antioxidants.

[70] Piao M.J., Ahn M.J., Kang K.A., Ryu Y.S., Hyun Y.J., Shilnikova K., Zhen A.X., Jeong J.W., Choi Y.H., Kang H.K. (2018). Particulate matter 2.5 damages skin cells by inducing oxidative stress, subcellular organelle dysfunction, and apoptosis. Arch. Toxicol..

[71] Bräuner E.V., Forchhammer L., Møller P., Simonsen J., Glasius M., Wåhlin P., Raaschou-Nielsen O., Loft S. (2007). Exposure to ultrafine particles from ambient air and oxidative stress-induced DNA damage. Environ. Health Perspect..

[72] Brunst K.J., Hsu H.L., Zhang L., Zhang X., Carroll K.N., Just A., Coull B.A., Kloog I., Wright R.O., Baccarelli A.A. (2022). Prenatal particulate matter exposure and mitochondrial mutational load at the maternal-fetal interface: Effect modification by genetic ancestry. Mitochondrion.

[73] Janssen B.G., Munters E., Pieters N., Smeets K., Cox B., Cuypers A., Fierens F., Penders J., Vangronsveld J., Gyselaers W. (2012). Placental mitochondrial DNA content and particulate air pollution during in utero life. Environ. Health Perspect..

[74] Wylie B.J., Matechi E., Kishashu Y., Fawzi W., Premji Z., Coull B.A., Hauser R., Ezzati M., Roberts D.J. (2017). Placental pathology associated with household air pollution in a cohort of pregnant women from dar es salaam, tanzania. Environ. Health Perspect..

[75] Jaligama S., Patel V.S., Wang P., Sallam A., Harding J., Kelley M., Mancuso S.R., Dugas T.R., Cormier S.A. (2018). Radical containing combustion derived particulate matter enhance pulmonary th17 inflammation via the aryl hydrocarbon receptor. Part. Fibre Toxicol..

[76] Aguilera J., Han X., Cao S., Balmes J., Lurmann F., Tyner T., Lutzker L., Noth E., Hammond S.K., Sampath V. (2022). Increases in ambient air pollutants during pregnancy are linked to increases in methylation of il4, il10, and ifnγ. Clin. Epigenet..

[77] Miyata R., van Eeden S.F. (2011). The innate and adaptive immune response induced by alveolar macrophages exposed to ambient particulate matter. Toxicol. Appl. Pharmacol..

[78] Parikh M.N., Brokamp C., Rasnick E., Ding L., Mersha T.B., Bowers K., Folger A.T. (2022). Epigenome-wide association of neonatal methylation and trimester-specific prenatal PM_2.5_ exposure. Environ. Epidemiol..

[79] Szpak D., Grochowalski A., Chrząszcz R., Florek E., Jawień W., Undas A. (2013). Tobacco smoke exposure and endothelial dysfunction in patients with advanced coronary artery disease. Pol. Arch. Med. Wewn..

[80] Fongsodsri K., Chamnanchanunt S., Desakorn V., Thanachartwet V., Sahassananda D., Rojnuckarin P., Umemura T. (2021). Particulate matter 2.5 and hematological disorders from dust to diseases: A systematic review of available evidence. Front. Med..

[81] Farahani V.J., Pirhadi M., Sioutas C. (2021). Are standardized diesel exhaust particles (dep) representative of ambient particles in air pollution toxicological studies?. Sci. Total Environ..

[82] Kim W., Cho Y., Song M.K., Lim J.H., Kim J.Y., Gye M.C., Ryu J.C. (2018). Effect of particulate matter 2.5 on gene expression profile and cell signaling in jeg-3 human placenta cells. Environ. Toxicol..

[83] Li S., Li L., Zhang C., Fu H., Yu S., Zhou M., Guo J., Fang Z., Li A., Zhao M. (2023). PM_2.5_ leads to adverse pregnancy outcomes by inducing trophoblast oxidative stress and mitochondrial apoptosis via klf9/cyp1a1 transcriptional axis. eLife.

[84] Alexandrova M., Manchorova D., You Y., Terzieva A., Dimitrova V., Mor G., Dimova T. (2023). Validation of the sw71-spheroid model with primary trophoblast cells. Am. J. Reprod. Immunol..

[85] Gupta S.K., Malhotra S.S., Malik A., Verma S., Chaudhary P. (2016). Cell signaling pathways involved during invasion and syncytialization of trophoblast cells. Am. J. Reprod. Immunol..

[86] Dietrich B., Haider S., Meinhardt G., Pollheimer J., Knöfler M. (2022). Wnt and notch signaling in human trophoblast development and differentiation. Cell Mol. Life Sci..

[87] Subbalakshmi A.R., Sahoo S., McMullen I., Saxena A.N., Venugopal S.K., Somarelli J.A., Jolly M.K. (2021). Klf4 induces mesenchymal-epithelial transition (met) by suppressing multiple emt-inducing transcription factors. Cancers.

[88] Thiery J.P. (2002). Epithelial-mesenchymal transitions in tumour progression. Nat. Rev. Cancer.

