# Supplementary figures and images for: Maternal Exposure to Diesel Exhaust Particles (DEPs) During Pregnancy and Adverse Pregnancy Outcomes: Focusing on the Effect of Particulate Matter on Trophoblast, Epithelial-Mesenchymal Transition
Source: Cells. 2025 Aug 26;14(17):1317. doi: 10.3390/cells14171317 (PMC12428444; doi:10.3390/cells14171317)

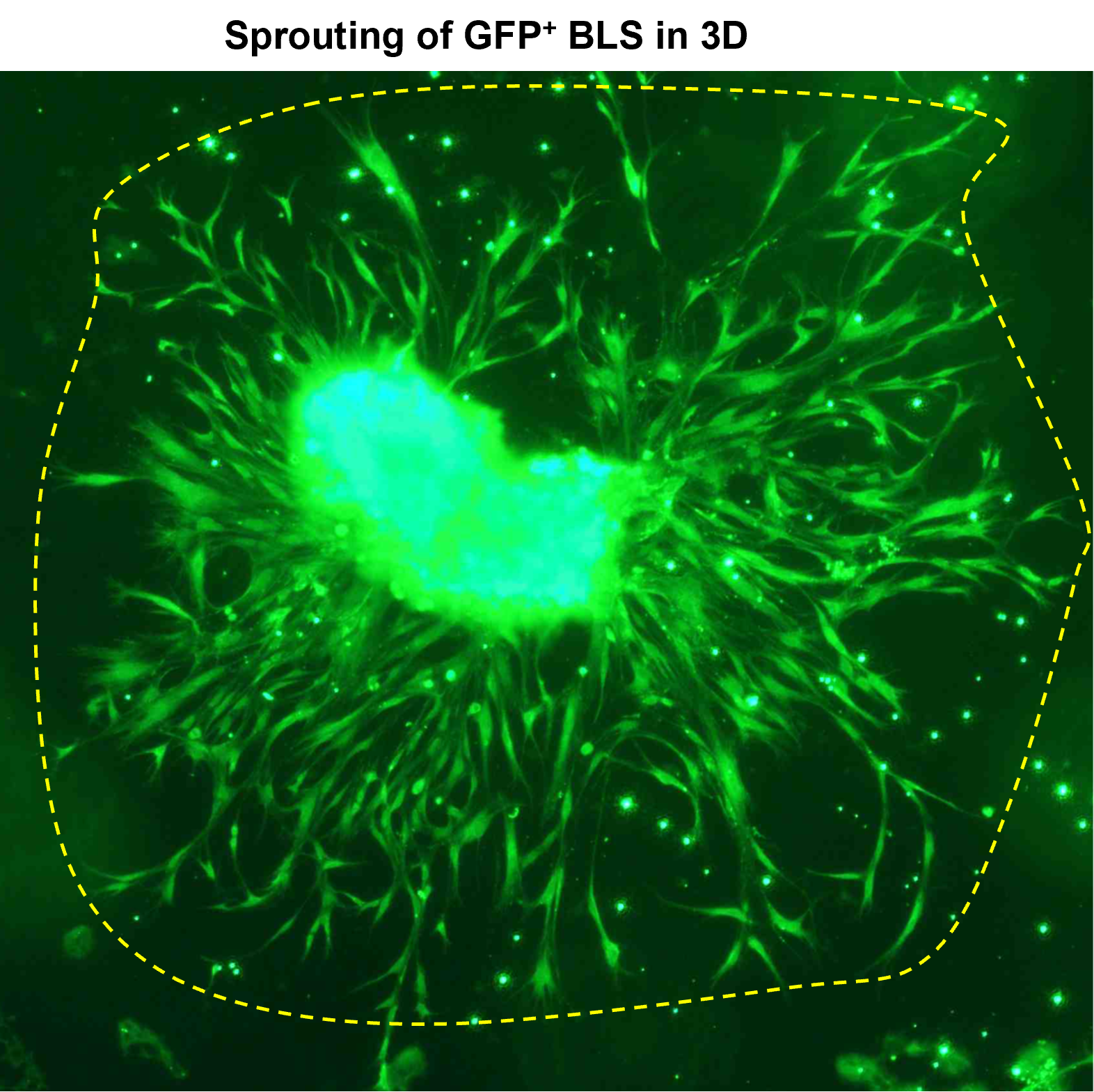

Supplement: Supplementary file 1 [file cells-14-01317-s001.zip › cells-3756138-supplementary.tif]
